# From sociolinguistic perception to strategic action in the study of social meaning

**DOI:** 10.1515/lingvan-2023-0183

**Published:** 2024-12-17

**Authors:** Gabriel Thiberge, Heather Burnett

**Affiliations:** Université Paris Cité, CNRS, Laboratoire de linguistique formelle, F-75013 Paris, France

**Keywords:** experimental sociolinguistics, social meaning, matched-guise task, video game

## Abstract

We present a new paradigm investigating social meaning through strategic action. More precisely, we present an experimental technique (a textual role-playing game developed with the Ren’Py engine), which we view as an enrichment of the matched-guise technique (MGT). In this paradigm the explicit response scales of the MGT are substituted for strategic choices in a video game. We argue that studying social meaning experimentally through looking at its effects on participants’ actions is more interactive than the classic paradigm. We compare the results from both the video game and a more classic version of the paradigm based on scales, conducted with the same linguistic materials, and we show that researchers who use only the MGT may be missing some crucial aspects of the social meanings of the linguistic phenomena they are studying. We also argue that a paradigm based on strategic action is better equipped to study the social, political, and economic outcomes for the users of those linguistic variants, and therefore to contribute to understanding phenomena like linguistic discrimination.

## 1 Introduction

This paper presents a new paradigm for studying *social meaning*. This term is used in different academic disciplines, often with different meanings. In sociolinguistics, it is often characterized as “the stances, personal characteristics, and personas indexed through the deployment of linguistic forms in interaction” (Podesva 2011: 234). Such definitions are based on the observation that listeners draw inferences about the properties of speakers depending on the language that they use. These inferences have been investigated experimentally in social psychology since the 1960s, in particular through using the matched-guise technique (MGT; Lambert et al. 1960). In the MGT, participants listen to samples of recorded speech or read short texts (called *guises*) that are designed to match as much as possible, differing only in the linguistic phenomenon studied. Each participant is exposed to only one of the guises, and after hearing it, their beliefs and attitudes towards the speaker are assessed, usually via questionnaire (for overviews, see Kircher 2015; Schleef 2022). The MGT was at first used to study listeners’ inferences about the characteristics of speakers of different languages; however, in the past 15 years, it has become widely used within variationist sociolinguistics to study subtle differences in the sociolinguistic perception of alternating linguistic forms, called *sociolinguistic variants* (Labov 1973; Tagliamonte 2012). For example, Campbell-Kibler (2007) used this paradigm to show that consistent associations exist between the variants -*ing* and -*in’* (i.e. alternative ways of pronouncing the final consonant in words like *working*) and the properties that listeners attribute to speakers who use these variants. Participants in Campbell-Kibler’s MGT study rated speakers as significantly more educated and more articulate in their -*ing* guises (i.e. when they said “working”) than in their -*in’* guises (i.e. when they said “workin”).

Although the linguistic phenomena studied using the MGT have evolved over the past 60 years, there has not been great evolution in the ways in which language attitudes or sociolinguistic perception are assessed. Although some studies have asked participants to freely provide adjectives describing the speakers in the different guises (Campbell-Kibler 2005), the heart of the output of a MGT study remains the scales upon which participants rate the speakers that they hear. As Kircher (2015: 200) describes, “the rating scales in matched-guise experiments tend to be interval, Likert-like scales with opposite extremes of certain traits at either end. Usually, half of these traits pertain to the status dimension and the other half of the traits pertain to the solidarity dimension.” This paper argues in favor of studying the effects of sociolinguistic variants on strategic action. More technically, we present an enrichment of the MGT, which substitutes the response scales for strategic choices in a video game, and we compare these results to those obtained in a more classic paradigm based on scales. Alongside a growing body of work examining how different experimental methods allow for an evaluation of implicit or explicit attitudes towards language variation (D’Onofrio 2018; Mack 2013; Pharao and Kristiansen 2019; Rosseel and Grondelaers 2019), we believe our new paradigm is both an indirect and ecological toolkit to assess how listeners build social representations of a speaker based on how they speak. By having speakers play what is presented as a real video game, we expect to get a clearer view of the associations between the social properties attributed to a person and their linguistic behavior.

More precisely, we argue that studying social meaning experimentally through looking at its effects on participants’ actions has three main benefits. First, the classic paradigm is a somewhat more implicit measure of social evaluation than, say, questionnaires or open interviews, and its quick replicability with multiple items and across numerous participants allows for a reliable measure of overall “group biases” (Lambert 1967). However, the task still relies on participants being able to verbally and explicitly describe their perceptions. This assumes that the mental representations triggered in sociolinguistic perception tasks happen to be well described by the particular predicates chosen for the scales, and that all participants interpret these predicates in the same way. While the success of the MGT suggests that at least some interpretations correspond well to predicates like *competent* or *laidback*, there is no evidence that all of them can be consciously accessed in the same way. Second, we argue that our new paradigm is more interactive than the classic paradigm. Indeed, as Kircher (2015: 205) says, a “criticism that has been brought forward against the matched-guise technique is that language attitudes that are elicited from ‘interactively non-involved’ participants are necessarily different from those of individuals actually participating in a particular speech exchange (Ryan et al. 1987, p. 1076).” Our strategic action paradigm places participants directly into an interaction, and our comparative study presented below shows that Ryan et al. (1987) are correct to worry about a gap between the sociolinguistic perception results (based on ratings) and the strategic action results (based on video games). We find that the strategic action results are more subtle: they show more fine-grained social meaning distinctions than the ratings. Finally, from a theoretical perspective, we argue that the strategic action paradigm can more directly express the relationship between language and the social order, in a more implicit approach that allows “linking the value and meaning of language to the value and meaning of the rest of the resources that count in society” (Heller and McElhinny 2017: xvi). Although the MGT allows researchers to get a glimpse of the kinds of ideologically important properties that participants associate with users of different linguistic variants, a paradigm based on strategic action is better equipped to study the social, political, and economic outcomes for the users of those linguistic variants, and therefore to contribute to understanding phenomena like linguistic discrimination (see Baugh 2017; Craft et al. 2020; Wright 2023) and commodification (see Duchêne and Heller 2012, among many others).

## 2 Video game study

### 2.1 Design and materials

**Figure 1:**
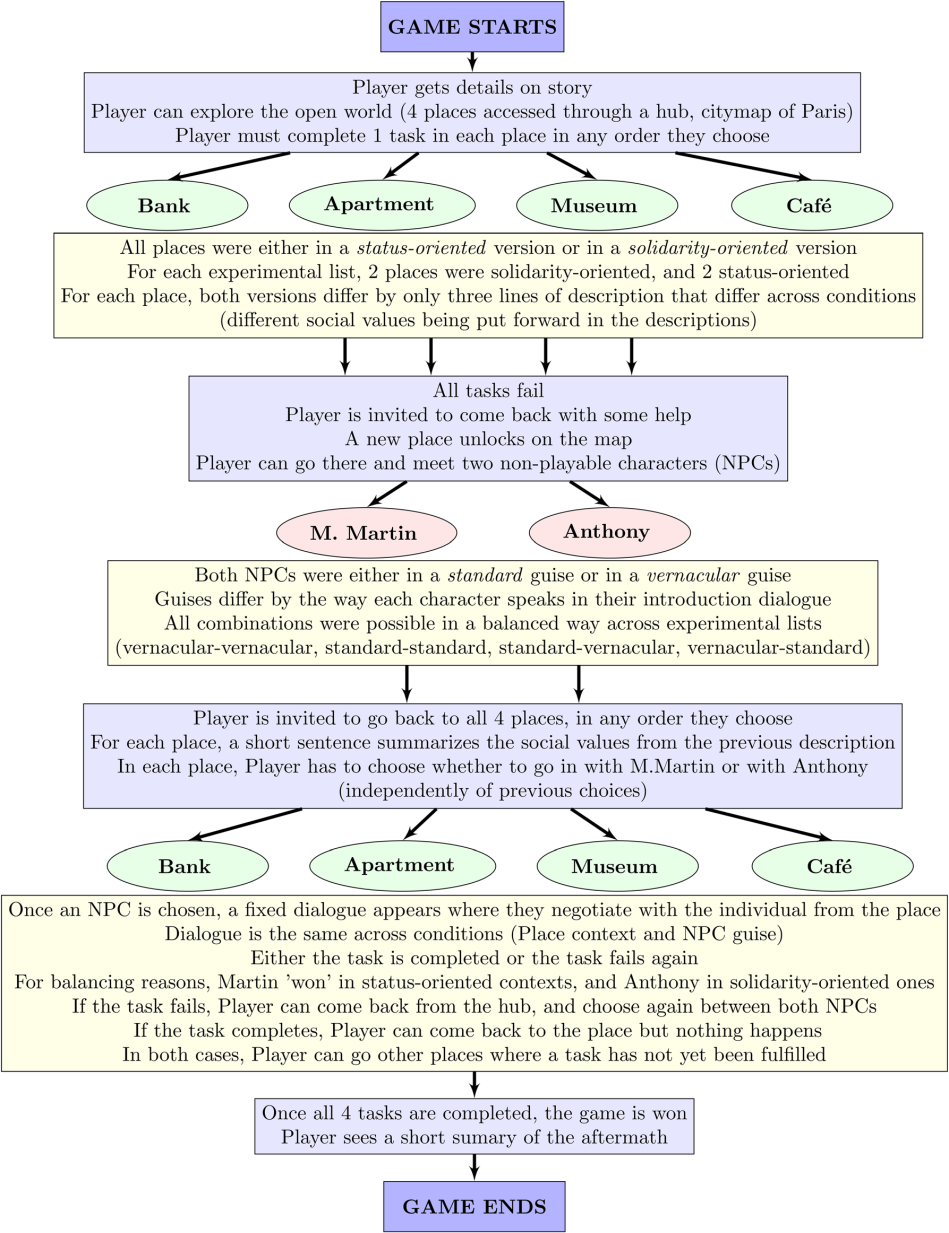
Flowchart of the game.

We developed a textual role-playing video game called *L’installation à Paris* ‘Moving to Paris’ in the Ren’Py engine (Rothamel 2022). A small open world was created around the city of Paris, where the player has to complete a series of quests (i.e. tasks) in order to stabilize their precarious situation: in the introduction to the game, it is explained that the player has recently arrived in the city and is sleeping on a cousin’s sofa. The player must complete four tasks: getting an apartment, opening a bank account, getting the attention of a server in a French café, and visiting a museum. At first the tasks cannot be completed, and the player has to explore and meet two non-playable characters (NPCs). Then, the player must request help from one of these NPCs in order to complete each task. To avoid biasing participants with nonlinguistic clues (clothing style, etc.), no visual indication of what the NPCs look like is ever presented, and only through the text can their personality, upbringing, and social status be imagined. One NPC was Monsieur Martin, an old-fashioned Parisian bourgeois who makes statements expressing *status* values: valuing family and tradition (Thévenot and Boltanski 1991; Woolard 1985). The other was Anthony, a down-to-earth middle-aged Parisian who makes statements valuing *solidarity*. Solidarity can be instantiated in concrete social interactions in many ways. Here, we took inspiration from Woolard (1985), who argues that the solidarity dimension is key for understanding sociolinguistic dynamics in situations of political conflict surrounding territory. We therefore had Anthony demonstrate solidarity values by resenting the gentrification of northeastern Paris and the recent displacement of less wealthy people from his neighborhood. A complete flowchart summarizes the course of a game in Figure 1.

**Figure 2:**
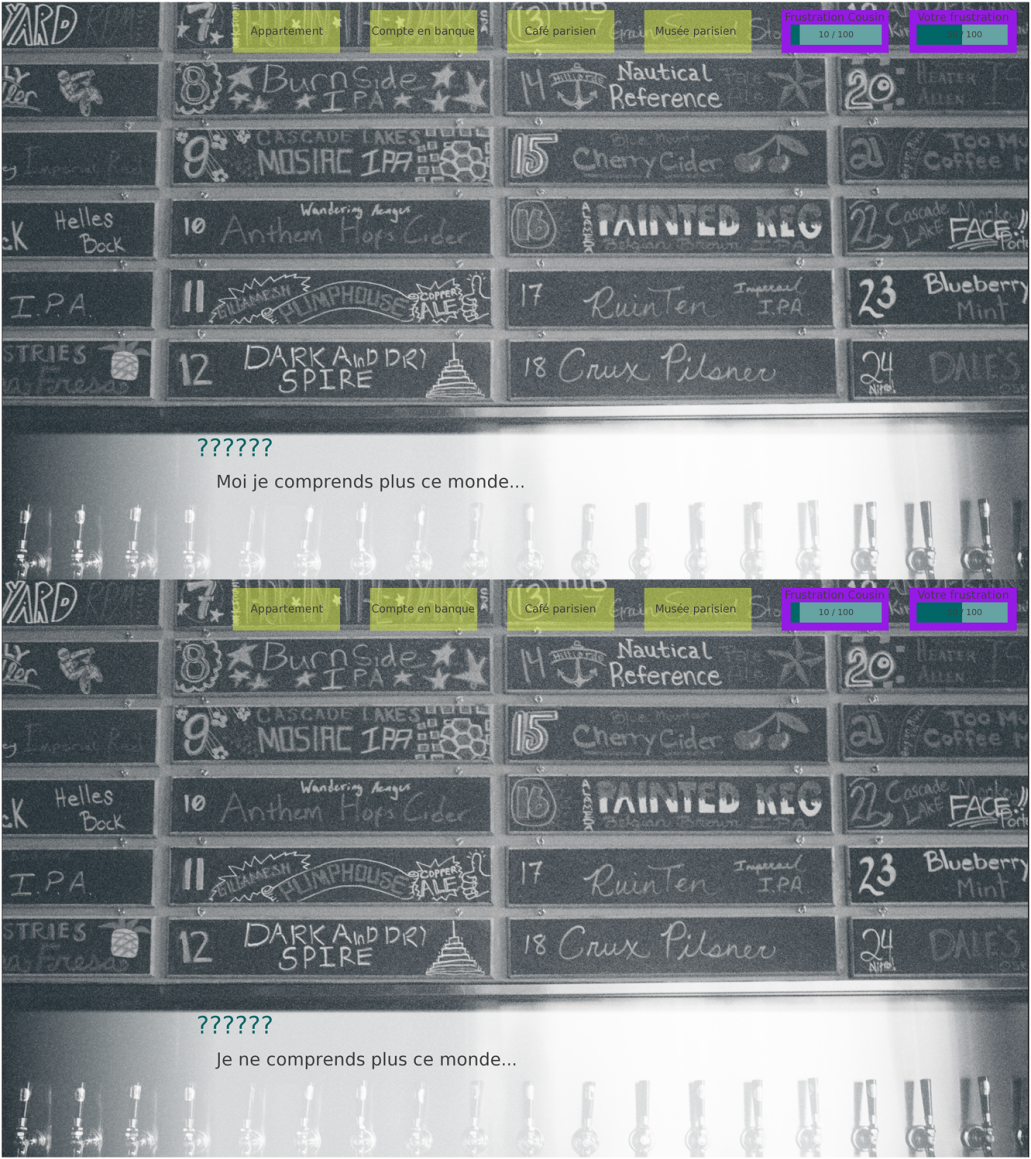
Screenshots of the same piece of dialogue from Anthony: ‘I don’t get this world anymore …’. *Top*, vernacular guise; *bottom*, standard guise.

The first manipulated experimental condition behind the game was the linguistic guise of each NPC, which was introduced during the presentation dialogue of each, in the location that unlocks after all tasks have failed once. In this dialogue, both NPC either used a “standard” or “prestige” variety of French (*standard guise*; Bourdieu and Boltanski 1975) or a “vernacular” or “covert prestige” variety (*vernacular guise*; Trudgill 1972). This dialogue was the only moment in the game where the NPC guises were accessible. Table 1 illustrates the linguistic points of variation between the two guises. All of these variables have been independently shown to be socially meaningful (Ashby 1977; Coveney 2005; Thiberge 2020). Figure 2 exemplifies how the two guises differed for different participants (here, during the introduction dialogue for Anthony).

**Table 1:** Points of linguistic variation in standard/vernacular guises.

Sociolinguistic variable	Guise	Example
Negative *ne* omission	standard	*Je* ***ne*** *le vois pas*
	vernacular	*Je le vois pas*
	translation	‘I don’t see him’
Subject doubling	standard	*Pierre est là*
	vernacular	*Pierre* ***il*** *est là*
	translation	‘Pierre is here’
Verb inversion in wh-questions	standard	*Combien* ***vendent*** *-ils ça?*
	vernacular	*Combien ils* ***vendent*** *ça?*
	translation	‘How much do they sell this for?’
Second person *tu/vous*	standard	*Voici* ***votre*** *journal*
	vernacular	*Voici* ***ton*** *journal*
	translation	‘Here is your paper’

**Figure 3:**
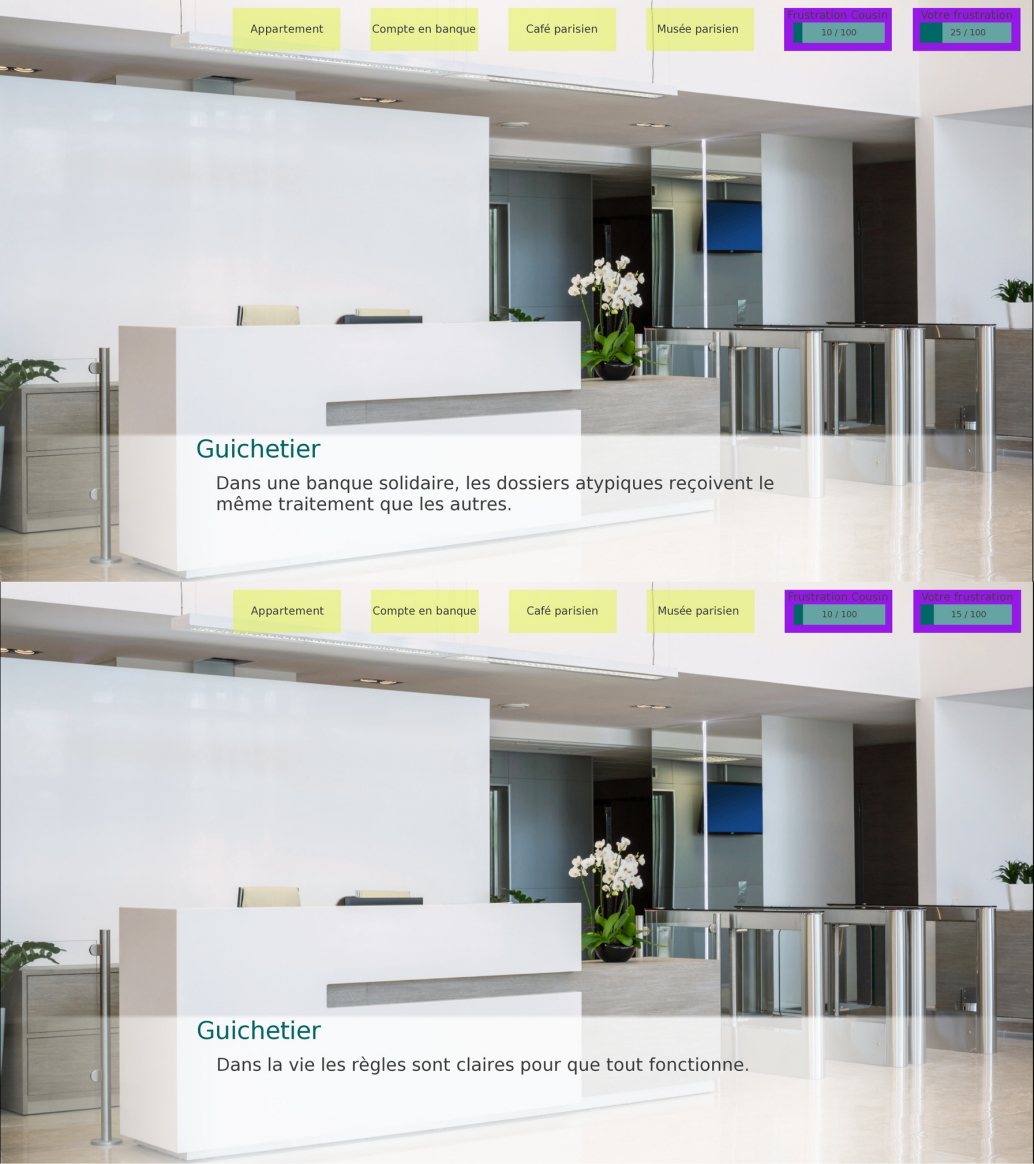
Two versions of the bank. *Top*, solidarity-oriented: ‘In a solidarity bank, atypical cases are treated the same as any other.’ *Bottom*, status-oriented: ‘In life the rules are clear so that everything can run smoothly.’

The second manipulated condition was the social context in which tasks had to be completed: all four places where the player had some quest pending also had two versions where different social values were put forward in the description. For instance, one player saw a *status-oriented* version of the bank, where financial stability and personal responsibility were explicitly expressed by a clerk, while another player saw a *solidarity-oriented* version of this location, where the clerk expressed solidarity and the need to take into account the very different personal situations of the clients. The “context” condition was laid out during the first time the player explored each location and before they failed each task, with three sentences alternating across conditions that described the social values important for each location. A one-sentence reminder of the social attributes for each context was presented to the player when they came back to the location with one of the NPCs to complete the task. Each player saw two status-oriented and two solidarity-oriented contexts (see Figure 3 for the two different versions of the bank location).

Due to the length of the game it is not possible to provide an exhaustive presentation of all the text shown to players during a game.1The game can be played at https://www.socialmeaning.eu/exp/gm/gm88/index.html (public, fully randomized version of the game). Participants had access to only one version of the game in a Latin square design, which allows for a good counterbalancing of experimental conditions. The textual descriptions of all four places and the guises of both NPCs can be found in the supplementary materials (https://osf.io/am5ur/).

### 2.2 Variables and predictions

Our independent variables are the guises of the NPCs and the status/solidarity-oriented versions of each location. Our dependent variable is the choice made by participants when they were prompted to select one of the two helping NPCs in each situation (recoded 0 = Anthony, 1 = Martin).

We only analyze here the first choice made by participants in the first situation. This amounts to 96 data points, which makes the dataset quite small. Our statistical analyses will thus use Bayesian logistic regression models rather than frequentist regression models, because Bayesian modeling is better suited for smaller datasets (Sorensen et al. 2016). Analyzing only the first choice excludes potential training effects from one quest to the next and does not take into account whether the choice allowed the quest to be completed or not. By “training effects” we mean that some participants may have worked out that two choices had to be “Martin” and two choices “Anthony” to win the game. Given that we did not constrain where participants made their first choice, and that they could fail multiple times in a row if they chose the same NPC in the same location over and over, players’ progression throughout the complete course of a game appeared too difficult to model.

Our predictions are based on existing sociolinguistics research, particularly classic works such as Bourdieu and Boltanski (1975), Woolard (1985), Labov (1973), and Trudgill (1972), and more recent experiments like Campbell-Kibler (2007), Podesva et al. (2015), and Beltrama et al. (2023):

– Prediction 1: Monsieur Martin expresses status values and will be preferred to Anthony in contexts valuing status. We assume that players will be (mostly) rational and choose the NPC that will give them the best chance to win the encounter. The solidarity-oriented NPC Anthony will be preferred in contexts valuing solidarity. However, we predict that the linguistic guises could perturb this pattern.
– Prediction 2: When Monsieur Martin is in his vernacular guise, we hypothesize that players’ preference for Martin in status contexts will decrease, compared to when he appears in his standard guise. Likewise, players’ preferences for Martin in solidarity contexts will be even more pronounced when Martin appears in his vernacular linguistic guise compared to his standard guise.
– Prediction 3: Conversely, we predict that Anthony will be even more preferred in solidarity contexts when he appears in a vernacular guise, compared to when he appears in a standard guise. Likewise, we predict he will be more preferred in his standard guise in status-oriented contexts than in his vernacular guise.

### 2.3 Participants and procedure

Ninety-six participants were recruited on Prolific (http://prolific.com). All were self-declared L1 adult speakers of French from France, with 48 over 30 years old and 48 under 30. They were asked to play the game, but nothing was made explicit about the experimental variables and conditions. Players were told they could explore the city through a clickable map of Paris and skip through text descriptions and dialogues via the space bar of their keyboard. They were told that they could go to several locations indicated by stars on the map, and that they would be prompted with text choices, without any indication as to whether these choices were good or bad, and only when a quest was finished did they see any form of progress. Players were informed that they had to complete all main quests for the game to finish.

Participants typically took 12–30 min to complete the game, with some outliers (10 min–1 h). Participants’ progression was tracked automatically in a new text file as soon as they started a game, with timestamps and important choices being recorded (i.e. did the participant select option 1 or option 2 at a particular choice point). Participation was anonymous, with a unique but unreadable ID attributed to every new game, in accordance with ethical guidelines.2IRB number: 00012023–23, CER Université Paris Cité. Participants received standard compensation (approximately £5 per 30 min).

Then we went through each text transcript of a game and coded all the meaningful choices that participants had made and all relevant data (sociolinguistic profile and experimental conditions).

### 2.4 Results

We analyzed our data with the R suite version 4.3.2 (R Core Team 2023) and RStudio (Posit Team 2023), within the Bayesian framework (logistic regression modeling; see supplementary materials for full specifications and the list of R packages we used).

#### 2.4.1 General results

**Figure 4:**
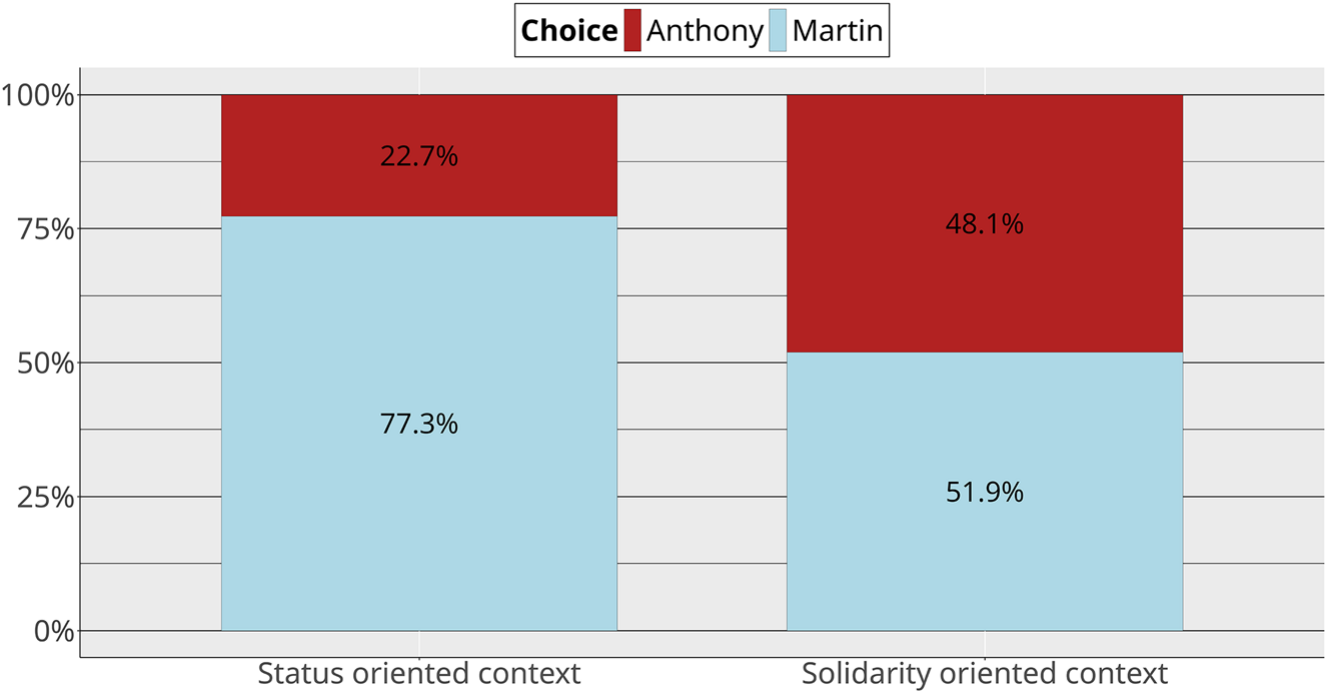
Choices by context (overall).

In line with prediction 1, we find that Martin is chosen more often in status-oriented contexts than in solidarity-oriented contexts (Figure 4). Anthony is chosen much more frequently in solidarity contexts than he is in status contexts, but Martin was still chosen (slightly) more than half of the time in solidarity contexts, which is surprising.

**Figure 5:**
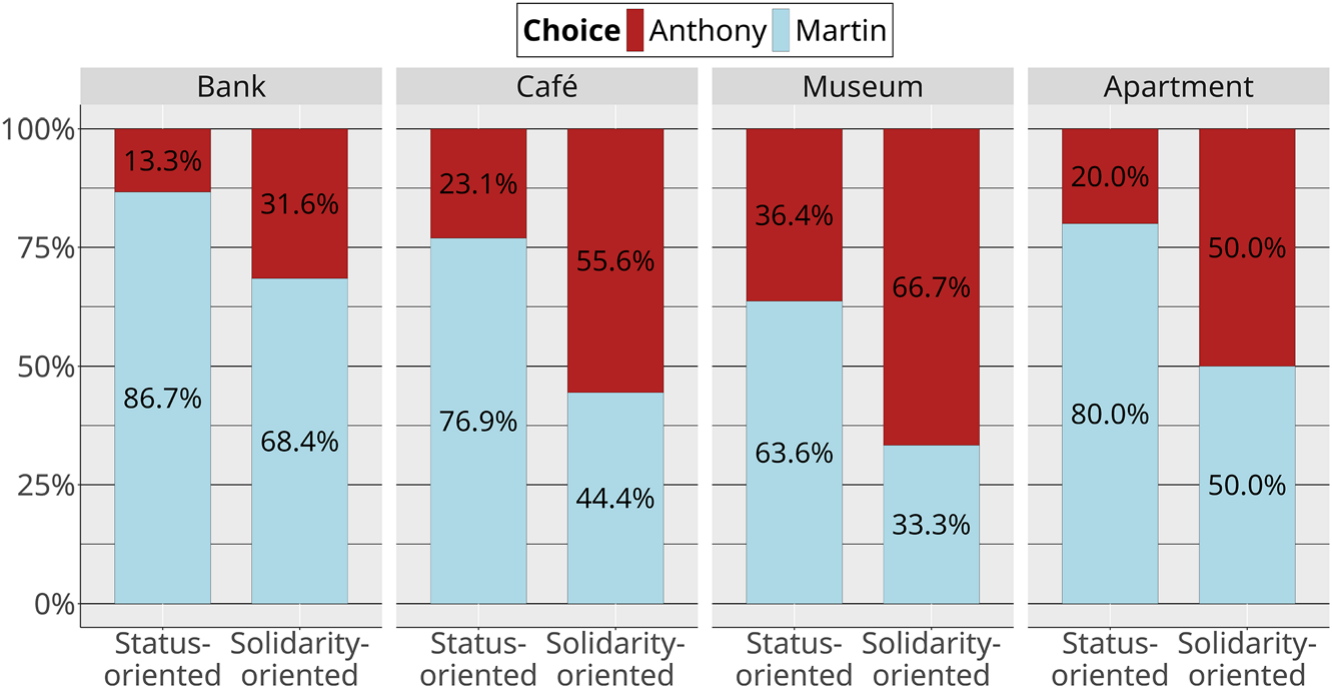
Choices by location and context.

However, further inspection of the different contexts shows that all status/solidarity contexts do not behave in the same way. For one thing, players’ first choices are not equally balanced across the bank (*n* = 34/96), café (*n* = 31/96), museum (*n* = 20/96), and apartment (*n* = 11/96), since we left players the freedom to explore our open world. Furthermore, the bank location, in both its status and solidarity versions, highly favored Martin (Figure 5). This is in contrast to the other locations, where the solidarity version favored Anthony (or there was no preference).

#### 2.4.2 Results: Martin’s guises

**Figure 6:**
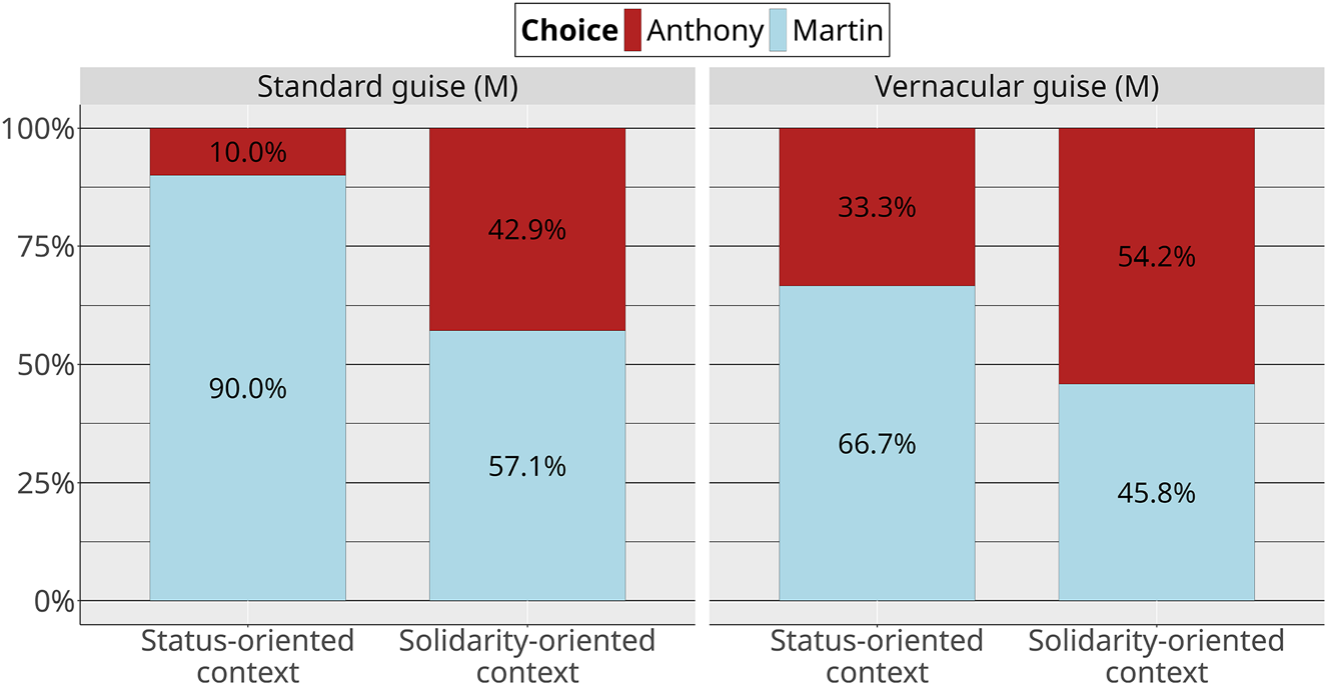
Choices by context and guise (Martin).

To analyze how this general pattern was affected by the linguistic guises of both NPCs, we first present how choices for Martin were related to his own linguistic guise (Figure 6).

With Bayesian modeling (model mb1.ctx.guiseM), we found several meaningful effects even with the relative scarcity of our data. First, there is robust confirmatory evidence for an overall effect of context: Martin was more chosen in status-oriented contexts (*β̂* = 1.66, 95 % CrI [0.60, 2.86], *P*(*β* > 0) = 1). Second, there is robust evidence for an overall effect of guise, with Martin being more chosen in his standard guise (*β̂* = 1.24, 95 % CrI [0.20, 2.38], *P*(*β* > 0) = 0.99). Third, we also find some evidence for an interaction between context and guise: the preference for Martin in status contexts over solidarity contexts is even greater when he appears in his standard guise than when he appears in his vernacular guise (*β̂* = 1.22, 95 % CrI [−0.88, 3.60], *P*(*β* > 0) = 0.86). In this way, the effects of the social meanings of the linguistic variants can be observed on the strategic choices another person will make in interaction with them.

It should be noted that these results do not capture the interaction between both NPCs guises, which we discuss below.

#### 2.4.3 Results: Anthony guises

**Figure 7:**
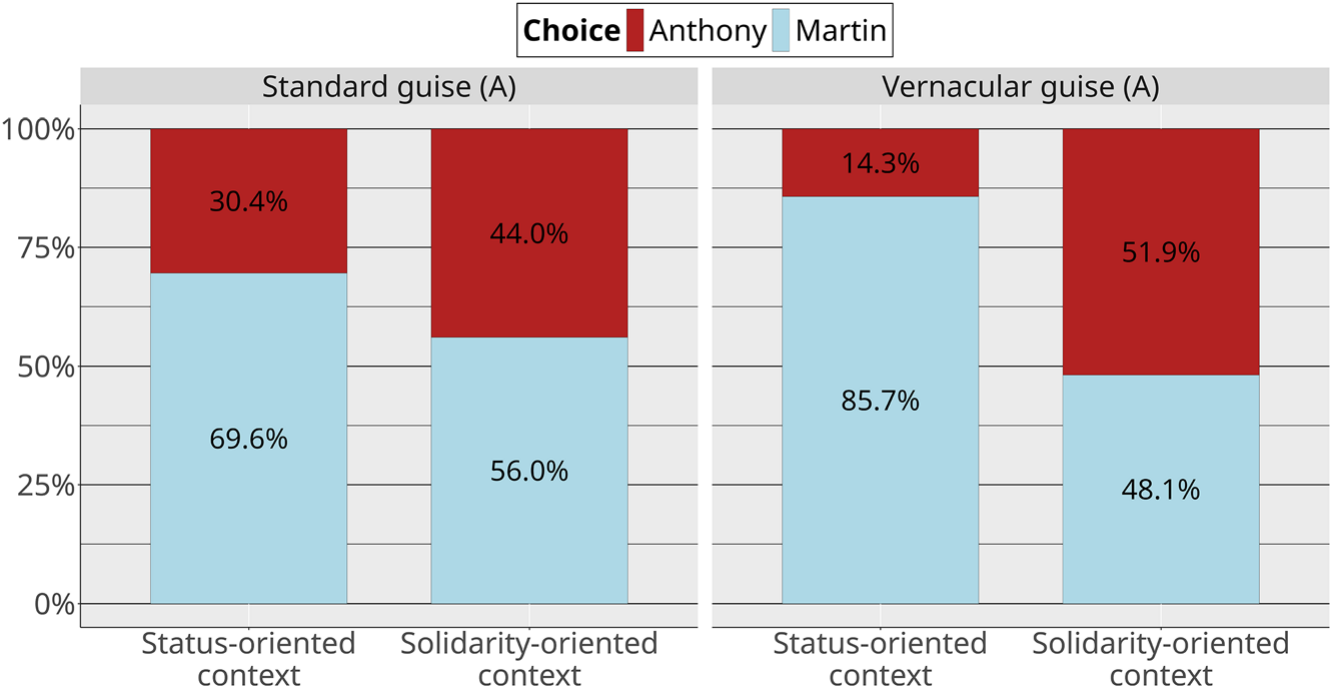
Choices by context and guise (Anthony).

Another way to look at the data is to look at how the choices for Martin were modulated by Anthony’s sociolinguistic variants (Figure 7).

In another model (mb1.ctx.guiseA, where the dependent variable was still the proportion of Martin choice), we find the same robust confirmatory evidence for an overall effect of context (Martin was more chosen in status-oriented contexts; *β̂* = 1.41, 95 % CrI [0.44, 2.45], *P*(*β* > 0) = 1). We also find robust evidence for an interaction between context and guise in this model (*β̂* = −1.46, 95 % CrI [−3.53, 0.50], *P*(*β* < 0) = 0.93). This means that Martin is more highly preferred in status contexts when Anthony is in his vernacular guise than when Anthony is in his standard guise. Put another way, Anthony is chosen more often in solidarity contexts when he is in his vernacular guise, and he is chosen more often in status-oriented contexts when he is in his standard guise. Again, these results do not yet include the possible interaction between NPCs guises.

#### 2.4.4 Results: interaction between guises

Since all four combinations of guises for NPCs were possible, a third model was run (mb1.ctx.guiseB) to account for a possible interaction between the guises and context. This model is more complex and the data is more stretched out across conditions, which makes for a more difficult extrapolation over the outputs. However it still supports the existence of all the effects mentioned above.

This model also yields robust evidence for an interaction Guise(Martin) × Guise(Anthony), independent of context, which points to more differences in the behavior of participants according to Martin’s guise, when Anthony was in his standard guise than when he was in his vernacular guise (*β̂* = −10.29, 95 % CrI [−45.52, 1.18], *P*(*β* < 0) = 0.92). This would indicate that standard Martin performs better than vernacular Martin against standard Anthony, while against vernacular Anthony both Martins hold pretty well.

Finally, this model yields robust evidence for a three-way interaction between context and both NPC guises (*β̂* = −27.78, 95 % CrI [−104.56, −2.84], *P*(*β* < 0) = 1). This points to a difference of the combined influence of NPC guises across contexts. When Martin is in his vernacular guise, there is not much of a difference in pattern between solidarity-oriented contexts and status-oriented contexts: Martin is a bit less chosen when Anthony is under his standard guise than when Anthony is under his vernacular guise. When Martin is in his standard guise however, the pattern is quite different across contexts: in solidarity-oriented contexts, Martin is more chosen when Anthony is under his standard guise, but in status-oriented contexts Martin is comparatively less chosen when Anthony is under his standard guise (and this is due to the fact that Anthony was never chosen when he was under his vernacular guise and Martin under his standard one).

### 2.5 Discussion

The results of the video game study show that linguistic variants can affect the strategic choices that participants make, opening up a new way to study their social meanings. Our predictions are mostly borne out. To begin with prediction 1, the nonlinguistic social properties conveyed by NPCs affected how they were chosen. Martin is chosen more frequently than Anthony in status-oriented contexts and, although Martin is still slightly preferred in solidarity-oriented contexts, Anthony is chosen much more frequently in solidarity-oriented contexts than in status-oriented contexts. The linguistic guises of both NPCs nuanced these general findings.

Prediction 2 was partially borne out: players had an even greater preference for Martin in status-oriented contexts when he appeared in his standard guise. However, he was still chosen more often in status contexts in his vernacular guise, suggesting that, while his language did modulate participants’ perceptions of him, using vernacular language over standard language was not sufficient to compensate for the status-oriented content of his speech.

Prediction 3 was borne out. Anthony is more preferred in solidarity-oriented contexts when he appears in his vernacular guise than when he appears in his standard guise. Likewise, he is more preferred in his standard guise in status-oriented contexts than in his vernacular guise.

From this, we conclude that studying social meaning through looking at strategic choice, as in our video game, can reveal a wide range of results that are consistent with sociolinguistic theories. We now compare these results with those of a more traditional matched-guise experiment.

## 3 Matched-guise experiment

We ran a text-based version of the classic matched-guise paradigm. Soukup (2013) references different variations of this paradigm (open-guise, verbal guise), but also written tasks. Although this modality is less often used, probably as a consequence of a long tradition of sociophonetic studies, we opted for it to have a closer comparison between with the video game (for which the stimuli were written). Some of the studies we based our predictions upon are also written MGTs (Beltrama 2018; Beltrama et al. 2023), and for example Thiberge (2020) conducted several MGTs on the same sociolinguistic phenomenon (alternating partial interrogative forms in French), with no apparent difference in evaluative patterns across modalities.

### 3.1 Design, materials, and participants

The experiment took place on a university-hosted instance of the IbexFarm platform (Drummond 2016). Forty-eight different self-declared adult L1 speakers of French from France (24 aged 30 or less, 24 older than 30) from Prolific read the textual3That is, there was none of the nonlinguistic information or gameplay decorum found in the video game itself. descriptions of the status/solidarity contexts and the dialogues featuring the standard/vernacular NPCs (Monsieur Martin and Anthony).

All these were condensed into one text for each context/NPC, and we presented them with a Latin square and randomized design. Participants saw only one version of each place and one guise of each NPC, with a balanced number of standard-guise versus vernacular-guise NPCs and of solidarity-oriented versus status-oriented locations (6 items by list, no fillers). Participants had to give their impression on how important they thought some properties were in the location or for the character: education, tradition, hierarchy, and speaking “good” French (all four being status features), and solidarity and social justice (both being solidarity features).4The sociolinguistic literature on standardization and normative language has identified a wide number of properties related to the status dimension. There have been far fewer investigations into the solidarity dimension; therefore, we felt less confident including as many solidarity properties as status properties. Participants answered on six corresponding 11-point slider scales (a frequent format in France, from school evaluations to general surveys, from 0 “not at all important” to 10 “very important”). We obtained 48 × 6 × 6 = 1728 answers in total. Considering the small number of items and the high variability we could expect on such social evaluations, we also opted for analyses with Bayesian models. Since our dependent variables were ratings on interval scales, we opted for cumulative-link models, best suited for ordinal data.

### 3.2 Results

**Figure 8:**
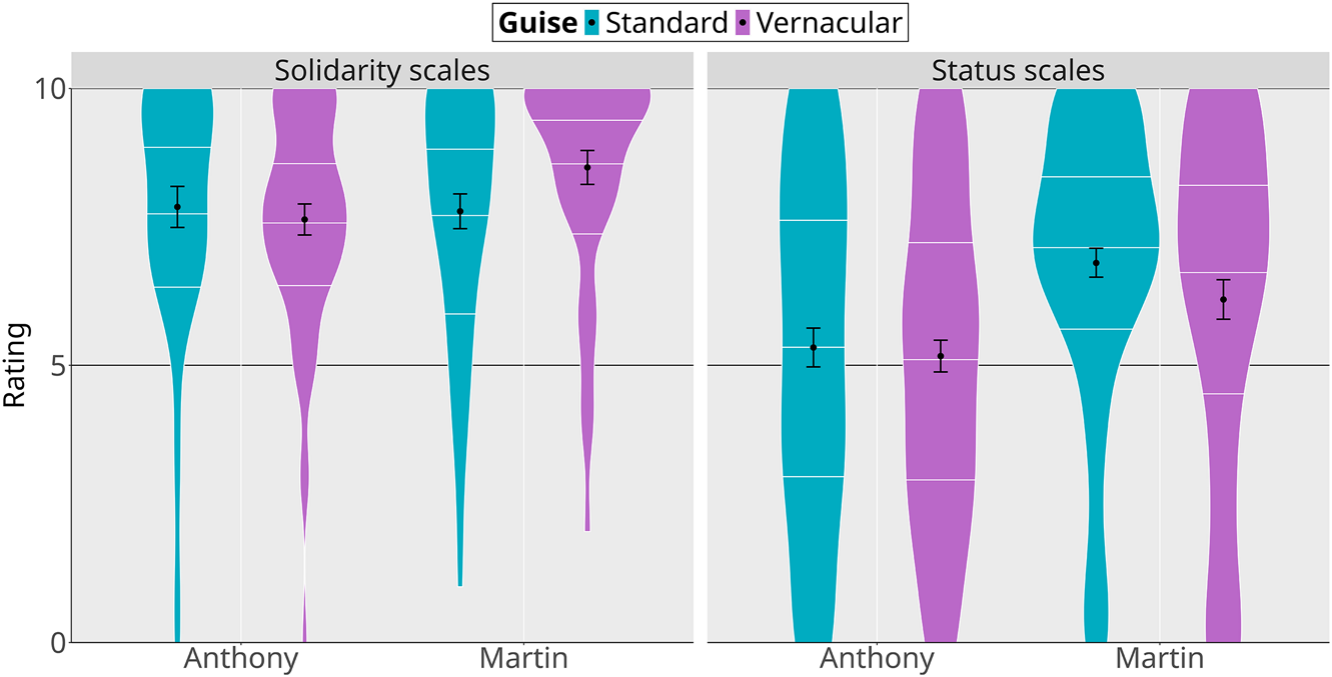
Matched-guise task results for non-playable characters (black dot: mean; error bars: standard error; white lines within the violins: quartiles).

Detailed analyses of the MGT results are provided in the supplementary materials (models mb1a.sopre for the NPCs, and mb1b.sopre for the locations). Figure 8 provides an overview of the social properties attributed to NPCs in their various guises, on the two kinds of scales (solidarity scales on the left, status scales on the right, mean and distribution of the ratings on the *y*-axis). On the solidarity scales, only Martin in his vernacular guise seems to yield higher results than all three other guises. In other words, the contrast between Anthony’s standard and vernacular guises that we observed in the strategic action paradigm was not observed in the MGT. On the status scales, we find a similar pattern, with Anthony showing no distinction between his two guises, although always being rated lower than Martin. Here again, it seems the contrast between the two linguistic guises that we observed in the video game is neutralized in the sociolinguistic perception task. On the other hand, the contrast between Martin’s two guises is observed in the MGT: as the sociolinguistics literature and the video game results would predict, Martin is rated higher on the status scales in his standard guise, and higher on the solidarity scales in his vernacular guise. This is captured in the model by the robust evidence for a three-way interaction between the NPC × scales × context variables (*β̂* = 1.54, 95 % CrI [−0.34, 3.49], *P*(*β* > 0) = 0.95).

**Figure 9:**
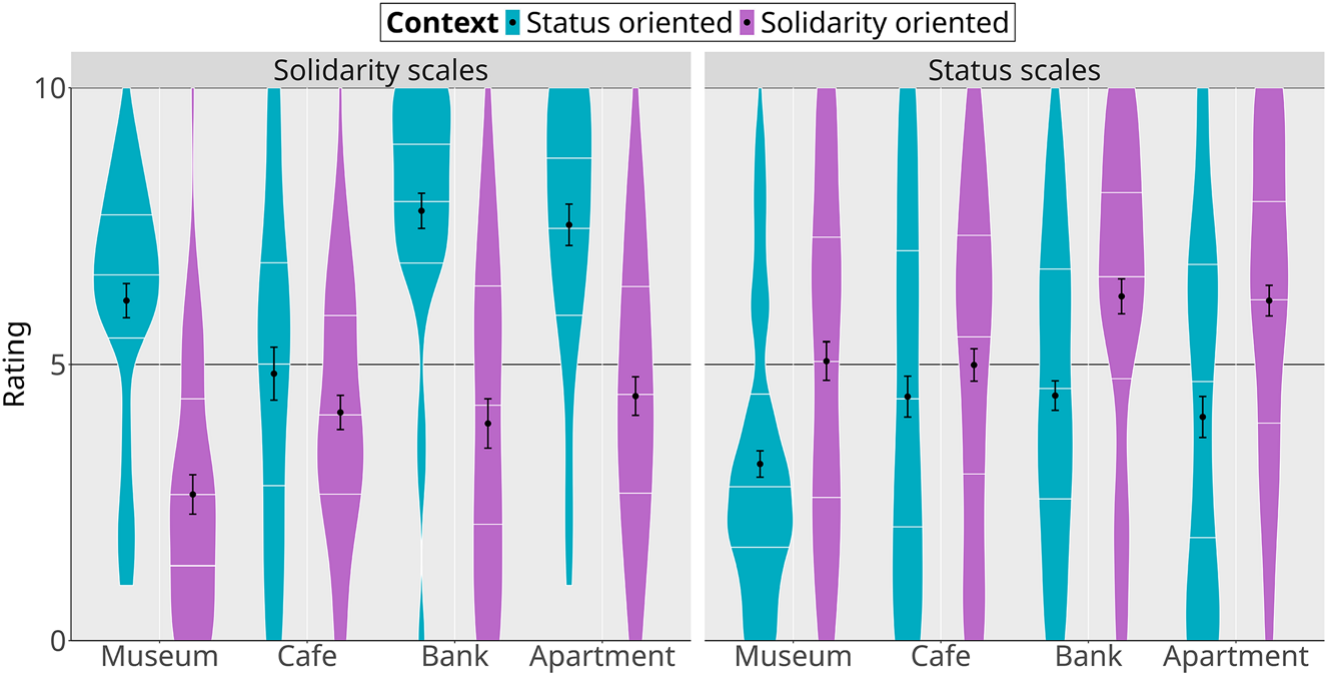
Matched-guise task results for locations (black dot: mean; error bars: standard error; white lines within the violins: quartiles).

We find a similar pattern for the contexts: as shown in Figure 9, the status and solidarity versions of each context do indeed rate higher on the status and solidarity scales, respectively. However, we again see some neutralization for the café location. Although there is a trend in the expected direction (status properties rated as less important in the solidarity-oriented café compared to the status-oriented version), we find robust evidence for a three-way interaction between the location × scales × context variables in a model with museum as the reference location (and solidarity scales and solidarity-oriented contexts as references for the two other variables; *β̂* = −2.91, 95 % CrI [−4.76, −1.03], *P*(*β* < 0) = 1). This indicates that the shift across scales is less important for the café than it is for the museum, bank, and apartment locations. Again, the lack of sharp differentiation in the MGT is surprising, because the two versions of the café are clearly distinguished in strategic action (Figure 5).

## 4 Discussion and conclusion

We compared the results of two experimental paradigms used to study social meaning: the matched-guise technique and a paradigm focusing on strategic action, as instantiated in a video game. We compared the language of two characters, M. Martin and Anthony; in particular, whether they used linguistic features from the French standard/prestige register or the nonstandard/vernacular register. We predicted that participants in the MGT study should rate both characters as higher on status scales when they appear in their standard guise, while rating them higher on solidarity scales when they appear in their vernacular guise. In parallel, we predicted that participants should be more likely to ask a character for help in the interactive video game in a status-oriented context when the character appears in their standard guise than when they appear in their vernacular guise, and vice versa with solidarity-oriented contexts.

More of the predictions were borne out in the strategic action study than in the matched-guise study. With the exception of vernacular Martin being chosen in solidarity contexts slightly less than we expected, the predicted patterns are all found in the video game. In the matched guise, however, some of these patterns are neutralized: for example, the matched-guise results make no distinctions between Anthony’s linguistic guises. At first glance, this pattern could be attributed to a “context dilution” effect, of the type observed by, for example, Hilton and Jeong (2019) and Pozniak et al. (2023), in which the perception of sociolinguistic variables in a matched-guise experiment is weakened in more detailed contexts, compared to in single sentences. However, we also saw the neutralization in the descriptions of the contexts, which did not vary sociolinguistically. In particular, there is no evidence that the status-oriented and solidarity-oriented versions of the café are different in the matched-guise results (Figure 9), despite them being clearly different in the video game results. This suggests that the neutralizations are most likely a product of the rating task in the MGT, rather than some property of sociolinguistic perception. In other words, when participants are forced to verbalize their social perceptions about a person or a place, the result appears to be less sensitive than when they are asked to interact with that person and/or in that place. Our results justify the concerns of Ryan et al. (1987) about the lack of interactivity in the matched guise: researchers who use only the MGT may be missing some crucial aspects of the social meanings of the linguistic phenomena they are studying.

Ratings on Likert scales appear to be blunter instruments for studying social meaning than video games, and we highlight that measuring the social meaning of a linguistic variant through looking at how it changes a participant’s strategy in an interactive game can bring experimental studies in closer contact with sociolinguistic investigations focused on how language affects speakers’ material (social, political, and economic) conditions. This by no means entails that the matched-guise technique should be abandoned, particularly given its relative ease of use and its proven track record. Rather, we argue that other paradigms, inspired by the MGT and used alongside the MGT, may help shed a different light on subtle phenomena rooted in sociolinguistic variation. The particular scenarios we investigated in our game concerned how language was related to certain areas of French administration, which is well known to be locus of the production and reproduction of social inequalities (L’Horty and Petit 2023). The strategic action paradigm, developed here using video games, is very general, and we believe that it could provide a more realistic yet controlled way to study a wide range of issues related to language and power, across contexts and cultures, in the future.
